# Evolution of drought and frost responses in cool season grasses (Pooideae): was drought tolerance a precursor to frost tolerance?

**DOI:** 10.1093/jxb/erae316

**Published:** 2024-07-27

**Authors:** Sylvia Pal Stolsmo, Camilla Lorange Lindberg, Rebekka Eriksen Ween, Laura Schat, Jill Christine Preston, Aelys Muriel Humphreys, Siri Fjellheim

**Affiliations:** Department of Plant Sciences, Norwegian University of Life Sciences, 1432 Ås, Norway; Department of Plant Sciences, Norwegian University of Life Sciences, 1432 Ås, Norway; Department of Plant Sciences, Norwegian University of Life Sciences, 1432 Ås, Norway; Department of Ecology, Environment and Plant Sciences, Stockholm University, SE-106 91 Stockholm, Sweden; Bolin Centre for Climate Research, Stockholm University, SE-106 91 Stockholm, Sweden; Department of Plant Biology, The University of Vermont, Burlington, VT 05405, USA; Department of Ecology, Environment and Plant Sciences, Stockholm University, SE-106 91 Stockholm, Sweden; Bolin Centre for Climate Research, Stockholm University, SE-106 91 Stockholm, Sweden; Department of Plant Sciences, Norwegian University of Life Sciences, 1432 Ås, Norway; University of Birmingham, UK

**Keywords:** Ancestral states, drought tolerance, ecophysiology, electrolyte leakage, frost tolerance, leaf dry matter content, phylogeny, Poaceae, Pooideae, precursor trait

## Abstract

Frost tolerance has evolved many times independently across flowering plants. However, conservation of several frost tolerance mechanisms among distant relatives suggests that apparently independent entries into freezing climates may have been facilitated by repeated modification of existing traits (‘precursor traits’). One possible precursor trait for freezing tolerance is drought tolerance, because palaeoclimatic data suggest plants were exposed to drought before frost and several studies have demonstrated shared physiological and genetic responses to drought and frost stress. Here, we combine ecophysiological experiments and comparative analyses to test the hypothesis that drought tolerance acted as a precursor to frost tolerance in cool-season grasses (Pooideae). Contrary to our predictions, we measured the highest levels of frost tolerance in species with the lowest ancestral drought tolerance, indicating that the two stress responses evolved independently in different lineages. We further show that drought tolerance is more evolutionarily labile than frost tolerance. This could limit our ability to reconstruct the order in which drought and frost responses evolved relative to each other. Further research is needed to determine whether our results are unique to Pooideae or general for flowering plants.

## Teaser

The highest levels of frost tolerance are found in species with the lowest ancestral drought tolerance, indicating that the two stress responses originated independently in different lineages of cool-season grasses (Pooideae).

## Introduction

Two-thirds of the global land area experiences frost at least some time during the year (Larcher, 2005). Frost is one of the most severe abiotic stresses plants can experience and the inability to cope with frost is thought to limit the distribution of many species (Donoghue, 2008). Based on this, it is widely held that evolutionary transitions from tropical, frost-free environments to those experiencing freezing are difficult. Despite this, temperate species are found in many angiosperm lineages (ca. 40% of families), and frost tolerance appears to have evolved multiple times independently (Ricklefs and Renner, 1994; Kottek *et al.*, 2006; Preston and Sandve, 2013; Stevens, 2017; Watcharamongkol *et al.*, 2018; Schubert *et al.*, 2020). One caveat of this view is that some cold stress responses are conserved across distantly related species and similar ancestral pathways have repeatedly been involved in their evolution (Preston and Sandve, 2013; Schubert *et al.*, 2019a). This suggests that the origin of frost tolerance in different lineages may not have been truly independent, but may instead have occurred by repeated modification of the same ancestral stress tolerance responses. Such ancient stress tolerance pathways may therefore have acted as precursors, or exaptations, to the sophisticated frost tolerance responses of many lineages today.

The most obvious candidate for an evolutionary precursor to frost tolerance is some form of drought tolerance. In general, strategies for avoiding dehydration are thought to be more ancient than adaptations to low temperature stress. All land plants need some basic mechanism for avoiding dehydration and some drought tolerance responses are ancient, most likely having their origins early during land plant terrestrialization or the evolution of vascular plants, some 400–500 million years ago (Mya) (Sakai and Larcher, 1987; Oliver *et al.*, 2000; Preston and Sandve, 2013; Bowles *et al.*, 2021). In contrast, while cool-climate pockets may have been present in Northern Hemisphere mid-latitude mountain areas in the Eocene (56–34 Mya; Hagen *et al.*, 2019), emergence of the cold and freezing environments of today is not thought to have begun until the late Eocene (mainly from ca. 34 Mya; Zachos *et al.*, 2001; Eldrett *et al.*, 2009; Liu *et al.*, 2009; Pound and Salzmann, 2017). Thus, flowering plants are thought to have evolved in a relatively warm world, with traits for dealing with frost stress evolving by independent repurposing of ancestral stress pathways (Preston and Sandve, 2013; Schubert *et al.*, 2019a, 2020).

The idea that there is a mechanistic link between adaptations to frost and drought was first put forward by Ebermayer (1873) in his ‘frost desiccation theory’. Ebermayer realized that both drought and frost stress require tolerance of cellular desiccation, and there is now ample evidence supporting the fact that the water deficit caused by both drought and freezing elicits many common physiological responses (Sakai and Larcher, 1987; Shinozaki and Yamaguchi-Shinozaki, 2000; Shinozaki *et al.*, 2003; Preston and Sandve, 2013). For example, both drought and frost can cause cells to collapse. Under freezing conditions, this is caused by ice crystal formation, either intracellularly, leading to mechanical puncturing of cell membranes, or extracellularly, leading to water withdrawal from the cells and causing them to shrink and collapse (Pearce, 2001). During drought, water deficit is the result of little to no available water or moisture. When the protoplast shrinks as a consequence of this, the concentration of cellular solutes increases above normal levels and, when the desiccation has reached a certain point, the cell collapses (Larcher, 2005).

Resistance to low water content in the cells can be induced by the synthesis and accumulation of solutes (Streeter *et al.*, 2001; Monson *et al.*, 2006), as well as through fortification and waterproofing of cell walls to protect the cell membrane against physical damage. These processes cause the intracellular water content to decrease (Vicre *et al.*, 2004), which increases the cells’ ability to maintain turgor at lower leaf water potential (Monson and Smith, 1982), leading to increased tolerance of both drought (Engelbrecht and Kursar, 2003) and frost (Anisko and Lindstrom, 1996). Furthermore, both accumulation of solutes and increased cell wall thickness raise the plant dry matter content; there is thus often a correlation between leaf dry matter content and resistance to desiccation (Cornelissen *et al.*, 2003). Accordingly, a positive relationship between leaf dry matter content and both frost and drought tolerance has been reported in various plants from different environments (Liu *et al.*, 2015; Pescador *et al.*, 2016). However, it is unclear whether high leaf dry matter content specifically confers tolerance of both drought and frost, or whether this is a general effect of greater desiccation resistance.

A physiological link between drought and frost stress has also been demonstrated in the field. Plants that have been exposed to drought and then subjected to frost show increased frost tolerance, whereas pre-treatment with heat had no effect on subsequent frost tolerance (Pisek and Larcher, 1954; Sumner *et al.*, 2022). Similarly, acclimation to freezing can result in acclimation to drought, and vice versa (Medeiros and Pockman, 2011; Hussain *et al.*, 2018), and plants from humid mountains often have lower frost tolerance than plants from arid mountains, even though the arid mountains are not necessarily colder than the humid ones (Sierra-Almeida *et al.*, 2016). Together, these studies suggest that stress pathways activated during one type of stress can yield physiological responses that are beneficial during the other. However, there has been little research addressing how the positive relationship between drought and frost responses evolved (see also Folk *et al.*, 2019, 2020).

Pooideae (Poaceae) are a globally distributed clade of cool season grasses dominating arctic, continental, and temperate floras (Hartley, 1973; Visser *et al.*, 2014; Zhang *et al.*, 2022). These habitats are characterized by short growing seasons, high temperature and precipitation seasonality, as well as episodic (short term/diurnal) and periodic (seasonal) frost and drought events. Pooideae are also distributed in a range of other environments, including forests, deserts and saline areas, with some lineages (e.g. Meliceae) being found in warmer and moister habitats and others (e.g. Triticeae) in colder and drier habitats (Bennett *et al.*, 2013; Kellogg, 2015; Zhang *et al.*, 2022). The global distribution of Pooideae across such divergent habitats makes the group well suited for testing how adaptations to dry and freezing environments evolved relative to each other.

The ancestors of Pooideae most likely evolved as forest understory plants during a warm period in the late Cretaceous (77–58 Mya) (Kellogg, 2001; Zachos *et al.*, 2001; Bouchenak-Khelladi *et al.*, 2010; Gallaher *et al.*, 2019, 2022; Schubert *et al.*, 2019b). It is thought that Pooideae transitioned from closed forest environments to open habitats several times independently, but it is unclear exactly how many times and in which lineages. Previous studies suggest at least two independent transitions to open habitats, in the most recent common ancestors of the core Pooideae (Poeae + Triticeae) and Stipeae, or in the lineages leading up to these clades (Zhang *et al.*, 2022; Elliott *et al*., 2024). Additional independent transitions may also have occurred in other lineages (e.g. in Nardeae and Lygeeae). These transitions began 67–58 Mya (Strömberg, 2011; Schubert *et al.*, 2019b; Gallaher *et al.*, 2022), with the most likely drivers being increased aridity and altered disturbance regimes, for example seasonality, fire and herbivory, rather than global cooling (Edwards *et al.*, 2010; Strömberg, 2011). Consequently, multiple lineages of Pooideae must have adapted to arid conditions early in their evolution.

It is unclear to what extent early Pooideae were exposed to cold conditions. Even though the climate was generally warm, previous reconstructions suggest that Pooideae originated in the Palaeoarctic, in cool-climate pockets in the emerging Eurasian alpine orogeny. They were therefore possibly exposed to short-term cool conditions, but not prolonged winters (Schubert *et al.*, 2019b; Gallaher *et al.*, 2022; Das *et al.*, 2023). However, there remains considerable uncertainty regarding the biogeographical origins and the level of cold exposure experienced by early Pooideae. What is more certain is that from ca. 34 Mya onwards, Pooideae experienced progressively cooler and drier conditions while temperate biomes expanded (Zachos *et al.*, 2001; Pound and Salzmann, 2017). Accordingly, Pooideae are known to have successively evolved phenological and physiological adaptations to frost and short growing seasons (Sandve *et al.*, 2011; Fjellheim *et al.*, 2014, 2022; McKeown *et al.*, 2016; Zhong *et al.*, 2017), allowing them to diversify in northern temperate regions (Kellogg, 2001; Bouchenak-Khelladi *et al.*, 2010; Schubert *et al.*, 2020).

Here, we ask whether drought tolerance could have acted as an evolutionary precursor to frost tolerance during the evolution of Pooideae, by combining ecophysiological experiments with reconstructions of how drought and frost tolerance responses evolved using comparative phylogenetic analyses. We have previously shown that sets of the same drought tolerance genes are expressed by distantly related species of Pooideae in response to short-term cold exposure (Schubert *et al.*, 2019a). Here we build on these findings to test the predictions that (i) drought and frost responses are positively correlated, (ii) leaf dry matter content is positively correlated with drought and/or frost tolerance, (iii) frost tolerant species are nested in ancestrally drought tolerant clades, (iv) frost tolerance evolved more frequently in ancestrally drought tolerant than drought sensitive clades, and (v) climate conditions in species’ natural habitats can explain variation in drought and frost responses. Contrary to predictions, we find that frost and drought tolerance responses are negatively correlated, with frost tolerance being highest in ancestrally drought sensitive clades. Further, we find that high leaf dry matter content is associated with drought tolerance but not frost tolerance, while climate of origin is largely unrelated to either drought or frost responses.

## Materials and methods

### Species selection

Sixty-two accessions representing 61 species were included in the experiment, sampled based on seed availability and their climatic, geographic, and phylogenetic diversity. Two accessions of *Phleum pratense* were included, one identified simply as *P. pratense* and one identified as *P. pratense* ssp. *nodosum*. The sampled species are mainly perennials and represent six of the ten tribes of Pooideae, including all of the major ones (Table 1) (Soreng *et al.*, 2017). Species names follow accepted names according to The Plant List (2013).

**Table 1.** Species analysed in the experiment

Number	Tribe	Accepted name in The Plant List (2013)	Species used for phylogenetic analysis	Seed ID	Source	Country
SR3	POE	*Poa trivialis*	*Poa pratensis*	18304,1	NGB	Finland
SR4	POE	*Deschampsia cespitosa*		11127,2	NGB	Norway
SR5	POE	*Poa alpina*		1197,2	NGB	Sweden
SR6	POE	*Phleum alpinum*		1342,3	NGB	Sweden
SR7	POE	*Lolium perenne*		4262,2	NGB	Norway
SR8	POE	*Dactylis glomerata*		7723,1	NGB	Norway
SR9	POE	*Poa alopecurus*	*Poa billardierei*	0662293	RBG Kew	Falkland Islands
SR10	POE	*Poa bulbosa*	*Poa annua*	0176493	RBG Kew	Jordan
SR11	POE	*Festuca pratensis*		0055789	RBG Kew	Switzerland
SR13	POE	*Sesleria autumnalis*		GRA3624	IPK	Germany
SR14	POE	*Vulpia myuros*		GRA2908	IPK	Spain
SR15	POE	*Phleum pratense*	*Phleum arenarium*	PI319076	Grin	Spain
SR16	POE	*Puccinellia distans*		PI502580	Grin	Russian Federation
SR17	POE	*Festuca rubra*		PI595056	Grin	Norway
SR18	POE	*Festuca arundinacea*		PI601418	Grin	USA
SR19	POE	*Phleum pratense*		PI321682	Grin	France
SR20	POE	*Holcus lanatus*		PI442500	Grin	Belgium
SR21	POE	*Festuca ovina*		PI676237	Grin	Germany
SR22	POE	*Cynosurus cristatus*		16615,2	NGB	Sweeden
SR23	POE	*Alopecurus pratensis*		13377,1	NGB	Norway
SR24	POE	*Lolium multiflorum*		13320,1	NGB	Denmark
SR25	POE	*Deschampsia atropurpurea*		—	Sampled in wild	Norway
SR26	POE	*Poa glauca*	*Poa palustris*	—	Sampled in wild	Norway
SR28	POE	*Anthoxanthum odoratum*		18256,2	NGB	Finland
SR29	POE	*Phalaris arundinacea*		4199.3	NGB	Norway
SR30	POE	*Calamagrostis purpurea*	*Calamagrostis canadensis*	2172.1	NGB	Norway
SR31	POE	*Agrostis canina*		4356,2	NGB	Sweden
SR32	POE	*Polypogon viridis*		0081773	RBG Kew	Lesotho
SR33	POE	*Helictotrichon pratense*		GRA513	IPK	Germany
SR35	POE	*Koeleria glauca*	*Koeleria albida*	W613215	Grin	Kazakhstan
SR36	POE	*Trisetum flavescens*		PI422495	Grin	Germany
SR37	POE	*Briza minor*		PI204410	Grin	Turkey
SR38	POE	*Briza media*		PI350681	Grin	Netherlands
SR39	POE	*Agrostis capillaris*		4209,2	NGB	Norway
SR40	POE	*Trisetum spicatum*		—	Sampled in wild	Norway
SR41	POE	*Agrostis mertensii*	*Agrostis vinealis*	—	Sampled in wild	Norway
SR43	TRI	*Elymus repens*		90282.2	NGB	Former Soviet Union
SR44	TRI	*Triticum turgidum*		22751,1	NGB	Sweden
SR45	TRI	*Aegilops triuncialis*	*Aegilops cylindrica*	AE1557	IPK	Unknown
SR47	TRI	*Hystrix patula*	*Elymus trachycaulus*	W649580	Grin	USA
SR48	TRI	*Hordeum jubatum*		—	Impecta	Unknown
SR50	TRI	*Dasypyrum villosum*		6594,1	NGB	Greece
SR52	TRI	*Agropyron cristatum*		90257,1	NGB	Former Soviet Union
SR54	BRA	*Brachypodium pinnatum*		PI440172	Grin	Russian Federation
SR57	MEL	*Melica nutans*		GRA512	IPK	Germany
SR58	MEL	*Glyceria striata*	*Glyceria fluitans*	W650682	Grin	USA
SR61	MEL	*Glyceria occidentalis*		Ames31334	USDA ISU	USA
SR62	STI	*Nassella hyalina*	*Nassella viridula*	PI 289543	Grin	Argentina
SR64	STI	*Stipa capillata*	*Stipa juncea*	ZA394	Jelitto Perennial Seeds	Unknown
SR65	STI	*Stipa pekinense*		ZA398	Jelitto Perennial Seeds	Unknown
SR66	STI	*Stipa gigantea*	*Stipa lagascae*	ZA400	Jelitto Perennial Seeds	Unknown
SR67	STI	*Stipa ichu*		ZA399	Jelitto Perennial Seeds	Unknown
SR70	STI	*Nassella tenuissima*		ZA407	Jelitto Perennial Seeds	Unknown
SR71	STI	*Nassella trichotoma*	*Jarava media*	ZA406	Jelitto Perennial Seeds	Unknown
SR73	STI	*Piptochaetium fimbriatum*	*Piptochaetium avenaceum*	0093527	RBG Kew	USA
SR79	STI	*Nassella cernua*	*Nassella clarazii*	0527992	RBG Kew	USA
SR82	STI	*Stipa calamagrostis*		GRA2848	IPK	Spain
SR89	STI	*Stipa caragana*	*Stipa barbata*	0775014	RBG Kew	Kyrgyzstan
SR92	LYG	*Lygeum spartum*		0185109	RBG Kew	Egypt
SR99	STI	*Nassella pubiflora*	*Nassella filiculmis*	PI478575	Grin	Peru
SR100	MEL	*Melica ciliata*	*Melica minuta*	PI494705	Grin	Romania
SR101	STI	*Piptatherum miliaceum*		PI207772	Grin	Israel

The table shows the experimental population number, tribe, accepted scientific name, species from Schubert *et al.* (2019b) used as phylogenetic placeholders in phylogenetic analyses, seed ID, seed source and the country of origin. Tribes are abbreviated: BRA, Brachypodieae; LYG, Lygeeae; MEL, Meliceae; POE, Poeae; STI, Stipeae; TRI, Triticeae.

### Germination and growth

The experiment took place in a greenhouse at Vollebekk, Ås, Norway (59°39ʹ42.4″N 10°45ʹ01.5″E) from 14 September to 14 December 2018. The greenhouse had an average temperature of 17 °C and long day conditions with 16 h of light. The light (200 μmol m^−2^ s^−1^) was a mix of natural light through the windows and light from metal halide lamps with both Philips MASTER HPI-T Plus (400W/645 E40 1SL) and Osram POWERSTAR HQI-BT (400W/ D PRO) light bulbs.

To promote synchronized germination, seeds were stratified in humid soil at 4 °C for 4 d and then transferred to 25 °C for 24 h, all in darkness. The seeds were then transferred to the greenhouse for germination. When plants were large enough (~5 cm, approximately 2–3 weeks after germination), single tillers were pricked out in 8 × 8 cm^2^ pots filled with standard potting soil (‘Gartner jord’, Tjerbo Torvfabrikk, Rakkestad, Norway). After pricking out, plants were watered once with fertilized water containing a mix of 800 g 100 l^−1^ YaraTera Kristalon Indigo (9% N + 5% P + 25% K, Yara, Oslo, Norway) and 600 g 10 l^−1^ YaraLiva Calcinit (15.5% N + 19% Ca, Yara), in a solution with a conductivity of 1.7 mS cm^−1^. Then, plants were grown for 2 weeks without fertilizer and then for one more week with daily watering with the fertilizer solution. Fertilization was done to ensure that plants were robust at the start of the experiment and nutrients were not limiting regrowth after treatment. Plants were randomly rotated among the tables every week.

The experiment was carefully designed to avoid pseudoreplication and unintentional spatial effects on plant responses (Rogers *et al.*, 2021). After the initial 3 week growth period, plants were randomly assigned to one of four treatment groups: (i) sudden frost at −1 °C, (ii) sudden frost at −3 °C, (iii) drought, and (iv) control. For most species, 10 individual plants served as experimental units in each treatment group, with four additional individuals designated for initial electrolyte leakage and four for initial leaf dry matter content measurements (Supplementary Table S1). This amounted to a total of 48 individuals per species being used in the experiment (Supplementary Table S1). Eighteen species had fewer than 48 individuals (*n*=29–47), which resulted in a total of 2870 individual plants in the experiment (Supplementary Table S1).

The plants were randomly distributed in trays. Both the drought and frost treatments started on 22 October 2018. Throughout the whole experiment, plants were rotated twice a week to mitigate spatial effects. All plants were kept in a single greenhouse room for the duration of the experiment, except during the freezing treatment, when several chambers were used. Plants from each species were distributed across different chambers during freezing treatment to mitigate possible chamber effects.

### Drought treatment

The drought treatment took place in the greenhouse at Vollebekk with the light and temperature conditions described above. Since species have different rates of water uptake (Taiz *et al.*, 2015) and the soil content might differ slightly between pots, soil moisture was measured in all pots during the drought treatment. The drought zone was defined as ≤5% soil moisture. A HH2 Moisture Meter (Delta-T Devices Ltd, Cambridge, UK) with a Wet-2-sensor was used to measure soil moisture by placing it in the soil. To avoid taking measurements in the holes in the soil left from the previous measurement, which could influence the moisture reading, repeat measurements in the same pot were taken on opposite sides. In the case of large variation between the two measurements, a measurement was taken at a third corner of the pot and the average was used.

Soil moisture was measured at the onset of the drought treatment and then every fourth day until the end of the treatment. To determine when the plants entered the drought zone, we obtained the soil moisture decline rate, estimated using the initial and last soil moisture measurement of ≤20%:


$$\begin{array}{l} Soil\;moisture\;decline\;rate\;\left(r\right)\;=\;\frac{MS-ML}{n} \end{array}$$ (1)

where MS is the starting moisture, ML is the last moisture recorded, and *n* is number of days.

The soil moisture decline rate was then used to estimate an approximate date when soil moisture was ≤5%:


$$\begin{array}{l} Remaining\;days\;until\;drought\;zone\;is\;reached\;=\;ML\;\%-r\frac{\;\%\;}{day}\;\times \;x\;day\;=\;5\;\%\; \end{array}$$ (2)

where *r* is the soil moisture decline rate found by using Equation (1) and *x* is the number of days until the species hits the drought zone. Plants stayed in the drought zone for 4–5 d.

After the end of the drought treatment, leaves of 10 individuals per species were harvested for conductivity measurements (see below) and the plants were watered and cut down to approximately 2–4 cm height. Regrowth was scored after 2 and 3 weeks (see below).

### Sudden frost treatment

The sudden frost treatment took place in frost chambers (Weiss Umwelttechnik GMBH, model KWP 1000/55-10DU-S) at ‘Centre for Plant Research in Controlled Climate’, Ås, Norway (59°40ʹ08.7″N 10°46ʹ07.6″E) without additional light other than from the windows in the chambers. Minimum temperatures for mild and severe sudden frost were −1 °C and −3 °C, respectively. Plants subjected to sudden frost were moved directly from the 17 °C greenhouse to the frost conditions without acclimation. Following the protocol of Alm *et al.* (2011), the starting temperature was set to 0 °C for 12 h and then lowered by 1 °C h^−1^ to the minimum temperature, where it was kept for 24 h. Then the temperature was increased by 1 °C h^−1^ back to 0 °C. The plants remained at 0 °C for 24 h (−1 °C treatment) or 28 h (−3 °C treatment). Thereafter, the plants were watered and placed in a room at +3 °C to thaw. Leaves from four individuals per species were sampled, and electrolyte leakage was measured (see below). After 24 h at +3 °C, plants were moved back to the greenhouse and cut down to approximately 2–4 cm in height. Regrowth was scored after 2 and 3 weeks (see below).

### Control

Sudden frost and drought treatments were carried out simultaneously, which allowed for the use of the same control for both treatments. Control plants were kept in the greenhouse, under the conditions described above. Plants were randomized across the tables and watered every week. Control plants were cut down to 2–4 cm, their regrowth was scored after 2 and 3 weeks, and their electrolyte leakage measured (see below).

### Ecophysiological measurements

#### Leaf dry matter content

To be able to correlate drought and frost tolerance responses with leaf dry matter content (LDMC), four individuals per species were used to measure LDMC on the same day as the drought and frost treatment started. Fresh aboveground biomass was weighed for each plant, before being placed in individual paper bags, dried in a Unitherm drying oven (Russell-Lindsey Engineering Ltd, Birmingham, UK) at 90 °C for 14 h and weighed again. LDMC was calculated as:


$$\begin{array}{l} LDMC\;=\;\frac{DW}{WW}\;\times \;100\;\%\; \end{array}$$ (3)

where WW is wet weight and DW is dry weight.

#### Electrolyte leakage and conductivity measurements

To assess the damage caused by the treatments, electrolyte leakage/conductivity was measured before and after the drought and frost treatments for all plants, including controls. When a cell becomes damaged, it will release electrolytes (Hincha *et al.*, 1987). Conductivity (mS) is a measure of amount of electrolytes released by a damaged leaf, and high conductivity indicates high cell damage. Approximately 1 cm^2^ of a representative leaf was cut and placed in a tube with 10 ml distilled water. The samples were shaken at room temperature for 10 h before the conductivity was measured with a CWO Volmatic Mesur EC (Senmatic A/S DGT Volmatic, Søndersø, Denmark). The conductivity of the shaken samples was then divided by the maximum conductivity:


$$\begin{array}{l} Percentage\;conductivity\;=\;\frac{CS}{CB}\;\times \;100\%\; \end{array}$$ (4)

where CS is conductivity after shaking and CB is conductivity after boiling. To obtain the maximum electrolyte leakage per species for comparison, leaf samples were then boiled at approximately 97 °C for 11 min and the conductivity was measured again when the tubes had cooled down to room temperature (25 °C). To get percentage conductivity after each treatment, Equation (4) was used per individual per species.

To see if the treatments had any effect compared with the control group, the following was used (Fujikawa and Miura, 1986):


$$\begin{array}{l} Percentage\;damage\;=\;\frac{100(\;\%\;CT-\%\;CC)}{100-\%\;CC} \end{array}$$ (5)

where %CT and %CC are the percentage conductivity obtained using Equation (4) for the treatment and control groups, respectively.

#### Fluorescence measurements

Fluorescence was measured on the control and drought plants before the start of the treatment and every fourth day until the plants had stayed in the drought zone for 4–5 d. The drought and control groups were measured on the same day for all species. Fluorescence measurements were carried out using FluorPen FP100 (Photon Systems Instruments, Drasov, Czech Republic) with the OJIP fluorescence transient analysis program. This program measures *F*_v_/*F*_m_, which represents the maximum quantum yield of photosynthetic efficiency in photosystem II. If the value of *F*_v_/*F*_m_ is low, it can indicate that the plant is damaged due to low photosynthesis (Gilbert and Medina, 2016). The measurements were taken in the middle of a representative leaf per plant. To ensure an accurate measure of photosynthesis and to avoid light contamination, plants were placed in a dark room for 25–35 min before the fluorescence measurements were made in the dark. The plants were transferred to the dark 3 h after dawn. The following was used to get the fluorescence of drought plants in relation to the control plants:


$$\begin{array}{l} Percentage\;\;fluorescence\;=\;\frac{F_{V}/F_{mFD}}{F_{V}/F_{mFC}}\times 100\;\%\; \end{array}$$ (6)

where *F*_v_/*F*_mFD_ is the last measurement of the plant in the drought zone for each species before it was cut and *F*_v_/*F*_mFC_ is the average measurements of the control for each species throughout the whole experiment.

#### Regrowth

Regrowth was assessed visually on a scale from 0 to 9, where 0 is dead and 9 is normal growth (Larsen, 1978). The following was used to obtain an estimate for the treatment plants in relation to the control plants:


$$\begin{array}{l} Percentage\;\;regrowth\;=\;\frac{RT}{RC}\;\times \;100\;\%\; \end{array}$$ (7)

where RT is the average regrowth per species after 2 and 3 weeks for the treatment: RT=(RT_2weeks_+RT_3weeks_)/2; and RC is the average regrowth per species after 2 and 3 weeks for the control plants: RC=(RC_2weeks_+RC_3weeks_)/2.

### Statistical and phylogenetic analyses

#### Phylogenetic data

To analyse the experimental results in an evolutionary framework, information on the phylogenetic relationships among species was taken from Schubert *et al.* (2019b). The phylogenetic tree was pruned to retain only the species included in the experiment. Species in the experiment but not in the phylogeny (20 species) were assigned to the tips of their closest relatives (Supplementary Dataset S1). Phylogenies by Hamasha *et al.* (2012) and Cialdella *et al.* (2007) were used to place species in tribe Stipeae, Grebenstein *et al.* (1998) was used for *Helictotrichon*, and Gillespie *et al.* (2007) for *Poa*. If no published phylogeny containing the species from both the experiment and its closest relative in the Schubert tree was found, the species in the experiment was assigned to a randomly selected tip within its respective genus (Supplementary Dataset S1). The species mean for each of the seven experimental variables (i.e. regrowth, fluorescence, and conductivity following drought treatment and regrowth and conductivity following frost treatments at −1 and −3 °C; Supplementary Dataset S1) was used to represent each species in the analyses described below. All analyses were done with RStudio version 1.1.383 (RStudio Team, 2016) and/or R version 3.5.2 (R Core Team, 2018).

#### Covariation and correlation among experimental variables

To visualize patterns of co-variation among the seven experimental variables (above and Supplementary Dataset S1), we used a principal component analysis (PCA), performed using *ggbiplot* (Vu, 2011). Next, to test which experimental variables are statistically correlated with each other, pairwise regressions were performed using Pearson’s correlation test and the function ‘cor.test’. We also tested for autocorrelation among the residuals and, if detected, instead used a phylogenetic least-squares regression (PGLS), implemented with the ‘pgls’ function in *caper* (Orme *et al.*, 2018). All pairwise trait combinations were tested.

#### Phylogenetic signal

To test whether closely related species showed more similar drought and frost responses than expected for a random sample of species, we estimated the phylogenetic signal (λ) of each trait. This information was used to select experimental variables for the evolutionary analyses below. Because principal component (PC) axes 2 and 3 showed interesting patterns suggesting co-variation among frost and drought tolerance responses, we also tested whether there was a phylogenetic signal in either of these variables. The phylogenetic signal was estimated by comparing the fit of different models with distinct assumptions for the variable Pagel’s λ (Pagel, 1999). The Brownian motion (BM) model assumes λ=1, i.e. that the observed trait variance is completely correlated with the phylogenetic distance among species. The ‘white-noise’ model assumes λ=0, that is, trait variance is independent of phylogeny. Finally, the ‘lambda’ model allows the value of λ to be estimated from the observed data, assuming a value between 0 and 1. The best model was determined based on the sample-size corrected Akaike information criterion (AIC; Akaike, 1974), using a difference in AIC≥2 to reject an inferior model (Anderson and Burnham, 2004). Models were fitted using *geiger* (Harmon *et al.*, 2008).

#### Choosing experimental variables as proxies for drought and frost tolerance

We used the results of the pairwise correlations and estimates of phylogenetic signal to select experimental variables as proxies for drought and frost tolerance for further evolutionary analysis. We selected variables that were significantly correlated with other variables and that showed phylogenetic signal, because they convey information about several experimental responses and are more evolutionarily relevant. In this way ‘conductivity following drought treatment’ was selected as a proxy for drought tolerance and ‘regrowth following the sudden frost treatment at −3 °C’ as a proxy for frost tolerance. LDMC was also analysed separately to test for an evolutionary correlation with each proxy for drought and frost tolerance.

#### Ancestral state reconstruction

To visualize how drought and frost responses have evolved in Pooideae, ancestral states for the two proxy variables plus LDMC were reconstructed using the level of phylogenetic signal for each trait found above. This was achieved with the BM model, having first rescaled the phylogenetic branch lengths according to the phylogenetic signal of the trait in question (Table 2). Ancestral states were reconstructed under maximum likelihood, using the ‘ace’ function in *ape* (Paradis and Schliep, 2018), and branches were rescaled using ‘rescale’ in *geiger* (Harmon *et al.*, 2008). Finally, the reconstructed ancestral states were visualized using *ggtree* (Yu *et al.*, 2017), *cowplot* (Wilke, 2019) and *ggplot2* (Wickham, 2016). Evidence for the expected patterns of drought tolerance evolving first and frost tolerance originating within ancestrally drought tolerant clades was assessed by eye.

**Table 2.** Akaike information criterion (AIC) values for the Brownian motion (BM), ‘white’ (phylogenetically independent), and ‘lambda’ models and the value of lambda (λ) inferred under the best fitting model(s)

	AIC			
Model/variable	BM	White	Lambda	λ (best estimate)
Sudden frost −1 °C				
Regrowth	−28	−51	**−61**	0.47
Conductivity	513	**447**	449	0
Sudden frost −3 °C				
Regrowth	31	19	**5**	0.47
Conductivity	561	**551**	**550**	0 / 0.63
Drought				
Regrowth	−50	**−64**	−62	0
Conductivity	640	**602**	**603**	0 / 0.11
Fluorescence	83	**38**	40	0
Principal component				
PC2	240	**211**	213	0
PC3	241	**193**	195	0
Leaf dry matter	343	327	**318**	0.45

The best fitting model(s) for each trait, with lowest AIC, is shown in bold. Models are considered indistinguishable if the difference in AIC<2.

#### Relating species drought and frost responses and leaf dry matter content to local climate conditions

Finally, we tested whether species’ drought and frost responses and LDMC correlate with the climatic conditions in each species’ native environment. Because drought and frost responses may not only reflect adaptation to the local climatic conditions but can also bear signatures of species’ evolutionary and biogeographical histories (Freckleton and Jetz, 2009; Humphreys and Linder, 2013; Coelho *et al.*, 2019; Lancaster and Humphreys, 2020), we included information on species’ phylogenetic relatedness and spatial proximity in these tests. We used generalized least squares models, in which the variance in a response variable is partitioned into phylogenetic, spatial, and independent components (denoted λʹ, φ, and γ, respectively) (Freckleton and Jetz, 2009). The phylogenetic (λ) and spatial (φ) components are estimated during model fitting using maximum likelihood. The relative contribution of phylogeny, λʹ, is calculated from the maximum likelihood estimates of λ and φ, as (1–φ)*×*(1–λ). A high value (approaching 1) of either λʹ or φ indicates a large effect of phylogenetic relatedness or geographical proximity, respectively. If the relative contributions of spatial and phylogenetic distances do not sum to 1, then the remainder of the interspecific variation is independent of either geographical proximity or phylogenetic relatedness and can be related to other explanatory variables, such as local climatic variation.

To include spatial and phylogenetic distances in the models, a spatial distance matrix was calculated using the ‘earth.dist’ function in the R package *fossil* (Vavrek, 2011) and a phylogenetic variance–co-variance matrix was calculated using ‘vcv.phylo’ in *ape*. We defined drought tolerance (‘conductivity following drought treatment’), frost tolerance (‘regrowth following frost treatment at −3 °C’) and LDMC as response variables. As predictors we used four temperature (bio1, bio4, bio5, and bio6) and four precipitation (bio12, bio13, bio14, and bio15) variables from WorldClim2 (Fick and Hijmans, 2017), based on geographical occurrence data obtained from the Global Biodiversity Information Facility (GBIF). The eight BioClim variables were chosen to represent annual average conditions (bio1, average annual temperature; bio12, annual precipitation), upper and lower extremes (bio5, maximum temperature of the warmest month; bio13, precipitation of the wettest month; bio6, minimum temperature of the coldest month; bio14, precipitation of the driest month), and annual variation (bio4, temperature seasonality; bio15, precipitation seasonality). Geographical data were compiled by GBIF.org (2022a) and Schat *et al.* (2024, Preprint) (Supplementary Dataset S1), supplemented with data downloaded directly from GBIF for three species [*Achnatherum calamagrostis*, GBIF.org (2022b); *Lolium arundinaceum*, GBIF.org (2022c); and *Lolium pratense*, GBIF.org (2022d)]. GBIF occurrence records were filtered following Schat *et al.* (2024, Preprint). From these we extracted the median latitude and longitude across each species range to represent the geographical centroids and median values for the BioClim variables to represent the climatic conditions in each species’ native range.

First, we fitted normal univariate linear regressions for each predictor and response variable to assess each climate variable’s effect on each predictor using the ‘lm’ function in R. Next, we fitted univariate linear regressions with variance partitioning into phylogenetic, spatial, and independent components to assess each climate variable’s effect on each predictor when phylogenetic and spatial effects are accounted for. Finally, we proceeded with multiple regressions including just the predictors with the strongest effects in the univariate tests and calculating the variance partitioning among phylogenetic, spatial, and independent components as before. The regression models with variance partitioning models were fitted using the ‘regress’ function in the R package *regress* (Clifford and McCullagh, 2006, 2014) and code from Cardillo and Skeels (2016). For the multiple regressions we used AIC scores to compare the fit of a full model (including the predictors and spatial and phylogenetic distances) with the fit of a series of reduced models (including any combination of phylogenetic distance, spatial distance, and predictors). We note that this approach does not allow estimation of how much of the *total* trait variance can be attributed to phylogenetic and spatial distances relative to the local climate (equivalent to the *R*^2^ for a linear regression; see Ives, 2018; cf. Lancaster and Humphreys, 2020), but it does allow assessment of the effect of the local climate when phylogenetic and spatial effects are accounted for.

## Results

### Variation in drought and frost tolerance responses

Almost all individuals showed full regrowth after sudden frost at −1 °C and the drought treatment, whereas there was much more variation in regrowth after sudden frost at −3 °C, including no regrowth at all (Supplementary Dataset S2). Conductivity following drought and sudden frost at −3 °C also showed a range of values, whereas most plants had low conductivity following frost at −1 °C. The maximum quantum yield after drought measured between 0 and 0.95, with most plants having intermediate fluorescence (mean 0.55, SD 0.29).

### Principal component analysis

**Fig. 1.**
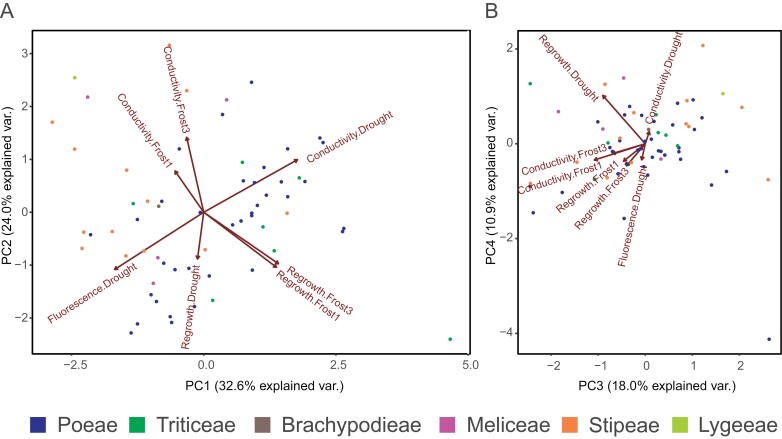
Principal component analysis (PCA) of the experimental variables. These plots are visualizations of the patterns of co-variation in the data, as a means of data exploration. (A) The first two principal components (PC1 and PC2), which together explain 57% of the variance. (B) The third and fourth principal components (PC3 and PC4), which together explain 29% of the variance. Each dot represents an accession/species (*n*=62), coloured according to the tribe in which it is classified. The arrows labelled with the experimental variables show in which direction and by how much (length of the arrow) each variable contributes to the distribution of the species, in relation to the other traits. The direction of each arrow in relation to each PC axis also shows which variables contribute most strongly to each PC. The seven experimental variables included are: regrowth, fluorescence, and conductivity following drought treatment and regrowth and conductivity following frost treatment at −1 and −3 °C (Supplementary Dataset S1). Overall, these plots show mixed patterns regarding how the drought and frost response measures co-vary with each other but species in the bottom left part of (A) show high tolerance of both drought and frost.

The PCA showed co-variation among several of the experimental variables (Fig. 1). The first four PCs explained 86% of the variance. Overall, there were clear patterns of co-variation among the different drought response measures and among the different frost response measures, but mixed patterns regarding how the drought and frost responses co-varied with each other (they do for PC2, partly for PC3, but not for PC1). PC1 explained 33% of the variance and primarily depicted the expected pattern of co-variation between conductivity and fluorescence following drought treatment (Fig. 1A). In addition, PC1 showed that drought tolerant species (low conductivity, high fluorescence) are less frost tolerant (low regrowth and high conductivity following frost treatment). PC2 (24% of the variance) had similar loadings from all experimental variables and showed that the three conductivity measures increase with increasing PC2, while all other measures decrease. Thus, species at the lower extreme of PC2 had high tolerance of both frost and drought. All the traits co-varied in the same direction with the third PC (18% of the variance; albeit with very low loadings for fluorescence and conductivity following drought treatment; Fig. 1B). Thus, lower extremes of PC3 mainly group frost tolerant species that are somewhat drought tolerant as well. The fourth PC (11% of the variance; Fig. 1B) mainly co-varied with regrowth after drought.

### Pairwise correlation tests

Five pairwise correlation tests were significant: regrowth after sudden frost at −1 °C and −3 °C (PGLS, *P*<<0.001, *R*^2^=0.32); conductivity after sudden frost at −1 °C and −3 °C (*P*<0.05, Pearson’s *r*=0.39); regrowth following sudden frost (−3 °C) and conductivity following drought treatment (*P*<0.05, Pearson’s *r*=0.27); conductivity and fluorescence following drought treatment (*P*<<0.001, Pearson’s *r*=−0.90); and LDMC and conductivity following drought treatment (PGLS, *P*<0.05, *R*^2^=0.16). Two further tests were marginally significant: regrowth following −1 °C and drought treatment (*P*=0.062) and LDMC and regrowth following −3 °C (*P*=0.072); we do not consider these tests any further. Thus, the only test suggesting a significant correlation between drought and frost responses indicated decreasing drought tolerance (increasing conductivity) with increasing frost tolerance (increasing regrowth). LDMC increased with increasing drought tolerance (decreasing conductivity) but showed no significant relationship with frost tolerance.

### Phylogenetic signal

The strongest phylogenetic signal was found for regrowth following frost treatment at both −1 and −3 °C (λ=0.47 in both cases) and LDMC (λ=0.45; Table 2). Some evidence for a phylogenetic signal was also found for conductivity following frost (−3 °C, λ=0.63) and drought (λ=0.11) treatment but the lambda and white models were statistically indistinguishable for these variables (Table 2). No other variable showed a phylogenetic signal. Frost treatment at −3 °C distinguished the species responses better than treatment at −1 °C, resulting in a response measure with greater variance. Therefore, conductivity following drought treatment and regrowth following frost treatment at −3 °C were used as proxies for drought and frost tolerance, respectively, in downstream analyses.

### Ancestral state reconstruction

**Fig. 2.**
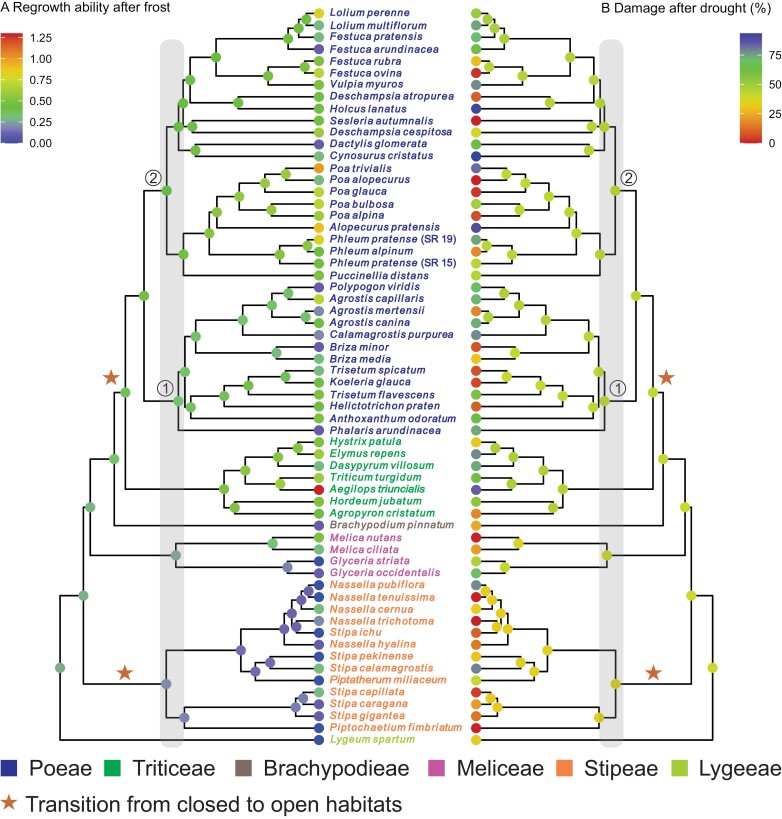
Ancestral state reconstructions of measured responses to frost and drought treatment based on a phylogenetic tree including all accessions/species in the experiment (*n*=62). (A) Regrowth ability following frost treatment at −3 °C. (B) Leaf damage (conductivity) following drought treatment. Values are expressed relative to the control. Node colours: reds indicate high levels of regrowth and low levels of damage/conductivity (i.e. high tolerance of frost and drought, respectively); blues indicate high levels of damage/conductivity and low levels of regrowth (i.e. poor tolerance of frost and drought, respectively). Overall, the ancestral state reconstructions show that high levels of frost tolerance (warmer colours, in A) evolved in clades that were ancestrally more drought sensitive (cooler colours, in B). Species names are coloured according to the tribe in which they are classified. Circled numbers indicate clades (‘chloroplast subgroups’) as defined by Soreng *et al.* (2017). Grey shading indicates approximately the Eocene–Oligocene boundary at 34 Mya (molecular dates from Schubert *et al.*, 2019b). Stars indicate putative transitions from closed to open habitats (Zhang *et al.*, 2022; Elliott *et al.*, 2024).

**Fig. 3.**
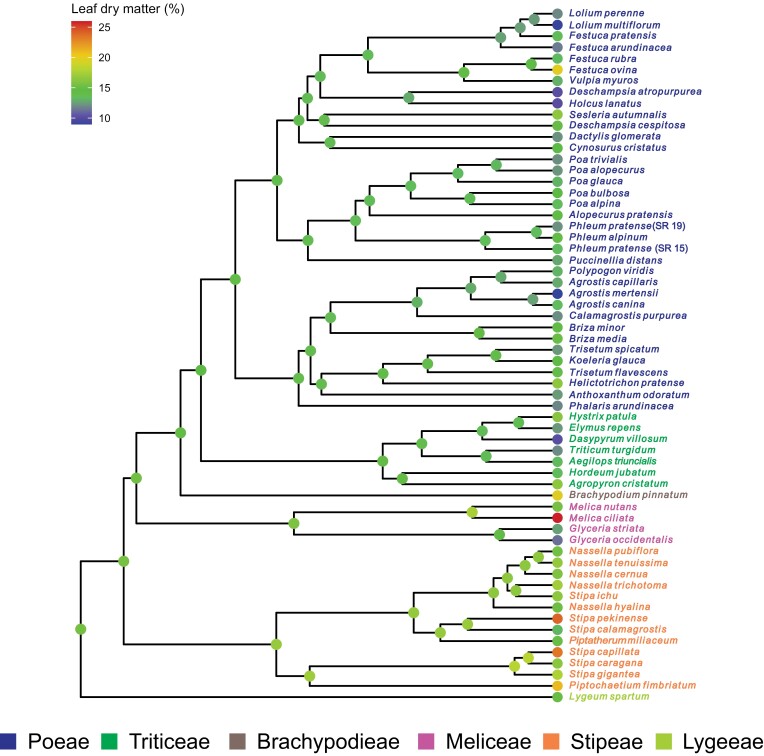
Ancestral state reconstruction for leaf dry matter content (LDMC) based on a phylogenetic tree including all accessions/species in the experiment (*n*=62). Node colours: reds indicate high LDMC; blues indicate low LDMC. Overall, the ancestral state reconstruction shows that the highest LDMC is found in clades that are the most drought tolerant, not frost tolerant (cf. Fig. 2). Species names are coloured according to the tribe in which they are classified.

The ancestral state reconstruction for drought tolerance showed that tribes Stipeae and Lygeeae were ancestrally more drought tolerant (lower conductivity; yellower internal nodes; Fig. 2), compared with the rest of the Pooideae (Meliceae, Brachypodieae, Triticeae, and Poeae; greener internal nodes; Fig. 2). Stipeae and Lygeeae were also inferred to have lower ancestral frost tolerance (less regrowth; blue internal nodes; Fig. 2) compared with the rest of Pooideae (green internal nodes; Fig. 2), with slightly higher ancestral frost tolerance in Triticeae and the Poeae chloroplast 2 clade (*sensu*  Soreng *et al.*, 2015, 2017) relative to other clades (yellower-green ancestral shades; Fig. 2). Finally, Stipeae and Lygeeae were inferred to have higher ancestral LDMC compared with core Pooideae (Poeae and Triticeae; Fig. 3).

### Spatial, phylogenetic, and climatic correlates of drought tolerance, frost tolerance, and leaf dry matter content

The univariate linear regressions suggested that average climate conditions across each species range are poor predictors of how species responded to the drought and frost treatments. For frost tolerance, one climate variable had a significant effect (Bio15, precipitation seasonality; *P*=0.032, *R*^2^=0.075), whereas for drought tolerance and LDMC none was significant (*P*>0.05). No adjustments for multiple testing were made.

The univariate models with variance partitioning into spatial, phylogenetic, and independent components revealed that phylogeny explained almost all of the variance for all three response variables (λʹ>0.999; Table 3). For frost tolerance, the strongest predictor effects were for three temperature variables (bio1, bio5, and bio6) but no test remained significant after correction for multiple testing (Table 3A). For drought tolerance, the strongest predictor effects were for three temperature variables (bio1, bio4, and bio6) and one precipitation variable (bio15), with all but bio1 remaining significant after correction for multiple testing (Table 3B). Finally, for LDMC, the strongest predictor effects were for one temperature (bio4) and two precipitation parameters (bio14 and bio15), but none remained significant after adjustment for multiple testing (Table 3C).

**Table 3.** Univariate linear models with variance partitioning into spatial (φ), phylogenetic (λʹ) and independent (γ) components and testing the effect of each BioClim predictor variable separately for frost tolerance (regrowth following −3 °C frost treatment), drought tolerance (conductivity following drought treatment) and leaf dry matter content

BioClim	Phylogeny (λʹ)	Spatial (φ)	Independent (γ)	Slope estimate	Slope standard error	*P*	P (Holm adjusted)
(A) Frost tolerance							
** bio1**	>0.9999	0.00	0.00	1.36	0.70	0.029	0.20
bio4	>0.9999	0.00	0.00	−0.0035	0.016	0.41	1.00
** bio5**	>0.9999	0.00	0.00	1.47	0.72	0.022	0.18
** bio6**	>0.9999	0.00	0.00	0.84	0.50	0.049	0.30
bio12	>0.9999	0.00	0.00	−0.017	0.016	0.15	0.59
bio13	>0.9999	0.00	0.00	−0.15	0.10	0.076	0.38
bio14	>0.9999	0.00	0.00	−0.079	0.25	0.38	1.00
bio15	>0.9999	0.00	0.00	−0.053	0.16	0.37	1.00
(B) Drought tolerance							
** bio1**	>0.9999	0.00	0.00	1.92	0.94	**0.022**	0.11
** bio4**	>0.9999	0.00	0.00	−0.071	0.020	**0.00032**	**0.0025**
bio5	>0.9999	0.00	0.00	0.072	0.99	0.47	0.65
** bio6**	>0.9999	0.00	0.00	1.85	0.64	**0.0027**	**0.019**
bio12	>0.9999	0.00	0.00	−0.017	0.021	0.22	0.65
bio13	>0.9999	0.00	0.00	0.072	0.14	0.30	0.65
bio14	>0.9999	0.00	0.00	−0.47	0.33	0.079	0.32
** bio15**	>0.9999	0.00	0.00	0.52	0.20	**0.0058**	**0.035**
(C) Leaf dry matter content							
bio1	1.00	0.00	0.00	−0.030	0.088569894	0.38	0.81
** bio4**	1.00	0.00	0.00	0.0032	0.001931794	**0.051**	0.31
bio5	1.00	0.00	0.00	0.056	0.090769901	0.27	0.81
bio6	1.00	0.00	0.00	−0.055	0.062410979	0.19	0.77
bio12	1.00	0.00	0.00	−0.000065	0.001961684	0.49	0.81
bio13	1.00	0.00	0.00	−0.020	0.012576533	0.061	0.31
** bio14**	1.00	0.00	0.00	0.058	0.029725438	**0.028**	0.20
** bio15**	1.00	0.00	0.00	−0.041	0.018356698	**0.015**	0.12

Significant tests are shown in bold.

The best multiple regression model for frost tolerance included just the predictors and phylogeny (ΔAIC≥12.6 compared with all other models; Table 4). Under this model, λʹ=0.50 and γ=0.50, meaning that half the variance is attributed to phylogenetic distance and half is independent of either phylogenetic or spatial distance. However, none of the predictors, bio1, bio5, and bio6, showed a significant effect (*P*=0.09, 0.08, and 0.08, respectively) but removing the predictors from the model completely led to a much worse model (ΔAIC=15.6; or ΔAIC=12.4 for the full model versus the spatial + phylogeny model; Table 4).

**Table 4.** Multiple regression models with variance partitioning into spatial (φ), phylogenetic (λʹ) and independent (γ) components and testing the effect of species’ local environment on measured (A) frost responses (regrowth following −3 °C frost treatment), (B) drought responses (conductivity following drought treatment), and (C) leaf dry matter content (LDMC)

	LL	AIC	ΔAIC	φ (spatial)	λʹ (phylogeny)	γ (independent)
(A) Frost tolerance						
Predictors + phylogeny + spatial	−225.7	465.3	27.0	0.00	>0.9999	<0.00001
Predictors + spatial	−220.5	452.9	14.6	0.00	—	1.00
** Predictors + phylogeny**	−213.2	**438.3**	**0.00**	—	0.50	0.50
Spatial + phylogeny	−237.6	483.2	44.9	0.00	>0.9999	0.0000006
Phylogeny only	−223.9	453.9	15.6	—	0.50	0.50
Spatial only	−233.0	472.0	33.7	0.00	—	1.00
Predictors only	−220.5	450.9	12.6	—	—	—
(B) Drought tolerance						
Predictors + phylogeny + spatial	−233.9	483.9	26.6	0.00	>0.9999	0.0000014
** Predictors + spatial**	−222.6	**459.3**	**2.00**	0.00	—	1.00
** Predictors + phylogeny**	−221.8	**457.6**	**0.30**	—	0.30	0.70
Spatial + phylogeny	−238.7	485.4	28.1	0.00	0.0000028	>0.9999
Phylogeny only	−237.8	481.6	24.3	—	0.19	0.81
Spatial only	−238.7	483.4	26.1	0.00	—	1.00
** Predictors only**	−222.6	**457.3**	**0.00**	—	—	—
(C) Leaf dry matter content						
Predictors + phylogeny + spatial	103.5	221.1	25.3	0.00	1.00	0.00
Predictors + spatial	−99.0	210.0	14.2	0.00	—	1.00
** Predictors + phylogeny**	−91.9	**195.8**	**0.00**	—	0.56	0.44
Spatial + phylogeny	−109.7	227.4	31.6	0.00	>0.9999	<0.0001
Phylogeny only	−97.0	200.0	4.2	—	0.49	0.51
Spatial only	−103.7	213.0	17.2	<0.0001	—	>0.9999
Predictors only	−99.0	208.0	12.2	—	—	—

Predictors refers to several bioclimatic variables (see ‘Materials and methods’ and Table 3). Best-fitting model(s) are shown in bold. AIC: Akaike information criterion; LL, log-likelihood.

For drought tolerance, three models were statistically indistinguishable from each other (0.30<ΔAIC<2.00; Table 4), the model including the predictors and just spatial distance, the model including the predictors and phylogenetic distance, and the model including only the predictors. However, under the spatial model, γ=1.00 (i.e. all variance is independent of spatial distance) and under the phylogenetic model, λʹ=0.30 and γ=0.70 (i.e. most variance is independent of phylogeny). Accordingly, the best model overall (albeit not significantly so) is the model including only the predictors (Table 4). Under this model, there is a significant effect of bio1 (*P*=0.035, slope=−6.64±3.59, *t*=−1.85) and bio6 (*P*=0.024, slope=7.36±3.65, *t*=2.02).

For LDMC, the best model was the one including the predictors and just the phylogeny (ΔAIC≥4.2, Table 4). Under this model, λʹ=0.56 and γ=0.44, meaning that just over half the variance is attributed to phylogenetic distance and the rest is independent of either phylogenetic or spatial distance. None of the predictors showed a significant effect (*P*>0.05). Accordingly, removing the predictors resulted in only a slightly worse model (ΔAIC=4.2).

## Discussion

### Present-day drought responses are negatively correlated with responses to episodic frost

In keeping with our predictions, we found that responses to drought and frost are correlated in Pooideae. However, in contrast to our predictions, the nature of this correlation shows that the species most tolerant of episodic (short-term) frost were the least tolerant of drought. This is evident from the pairwise correlations among the experimental variables and the PCA, which showed that species with high levels of damage following drought treatment were the least damaged by the frost treatments (Fig. 1). We assessed frost tolerance using the whole-plant responses survival and regrowth. However, because all species grew well following drought treatment, we were not able to use similar whole-plant responses for drought tolerance. Instead, we used electrolyte leakage. Previous studies have shown that this is a good proxy for drought tolerance measured as survival and regrowth (Bajji *et al.*, 2002). Therefore, the different measures of drought and frost responses are comparable. Furthermore, we found negative correlations between electrolyte leakage (conductivity) and photosynthetic capacity following drought treatment and between electrolyte leakage and regrowth following frost exposure at both −1 °C and –3 °C. The PCA plots also show co-variation among different measured responses to frost and drought treatments, respectively (Fig. 1). Thus, there are expected and reliable signals in our experimental data, meaning that the negative correlation between frost and drought tolerance found is unlikely to be an artefact of the experimental variables used. Instead, these results likely reflect different adaptations to different environments in different species.

Most species of Pooideae experience frost during their growing season (Schubert *et al.*, 2020), and there are clear physiological links between drought and frost stress, fuelling our hypothesis of their shared evolutionary origins (Sakai and Larcher, 1987; Shinozaki and Yamaguchi-Shinozaki, 2000; Shinozaki *et al.*, 2003; Preston and Sandve, 2013). Tolerance is, however, not the only strategy plants use to cope with stressful conditions. Plants can also escape damage from freezing or drought, e.g. by shedding leaves and becoming dormant, or through adopting an annual life history strategy and persisting as seed during adverse conditions. It would be interesting to determine whether adopting an annual life history strategy provides an advantage to Pooideae species in escaping both drought and frost, although initial investigations suggest that it does not (Lindberg *et al.*, 2020; Hjertaas *et al.*, 2023). Moreover, it should be noted that the responses we have measured likely represent several physiological stress responses. Further disentangling different physiological response mechanisms in future studies may provide additional insight into the evolution of the known overlap between drought and frost responses.

### Is leaf dry matter the link between frost and drought tolerance?

We found a significant negative relationship between leaf dry matter content and electrolyte leakage in response to drought, indicating that the former may confer drought tolerance in Pooideae. These results are in line with Liu *et al.* (2015), who found leaf dry matter content to be positively correlated with drought tolerance in *Magnolia*. We did not, however, find any relationship between leaf dry matter content and regrowth following frost treatment. This indicates that leaf dry matter content is not a component of short-term frost responses in Pooideae. This contrasts with Watcharamongkol (2019), who found a correlation between episodic frost tolerance and water content (the inverse of dry matter content) in the predominantly (sub)tropical PACMAD grasses. One explanation for these contrasting results could be that if high dry matter content was an exaptation to frost tolerance, such that it facilitated adaptation to freezing climates in already drought tolerant plants, the signature of that exaptation may be masked by the more sophisticated and complex freezing adaptations of present day temperate clades like Pooideae (Schubert *et al.*, 2019b). Thus, it is still possible that the first responses to episodic frost in an ancestor of Pooideae utilized early desiccation tolerance traits, including high dry matter content. Further study of the role of leaf dry matter content in the evolution of drought and frost responses at broader phylogenetic scales and tests for shared gene expression patterns linked to early dehydration traits, including dry matter content, is needed.

### Evolutionary trajectories of drought and frost responses

If drought tolerance was a precursor for frost tolerance, we would have expected drought tolerance to have evolved first in lineages tolerant of episodic (short-term/diurnal) frost, and/or frost tolerance to have evolved more frequently in ancestrally drought tolerant lineages. This is not what we found (Fig. 2). Instead, we found a mirrored phylogenetic pattern for drought and frost tolerance, with the lineage with the highest inferred ancestral drought tolerance (Stipeae) being the least frost tolerant and the highest frost tolerance being measured for species with the lowest ancestral drought tolerance (Poeae + Triticeae). This would suggest that the sophisticated drought and frost tolerance responses of extant species have originated as the result of independent evolutionary trajectories. These results corroborate findings from a comparative analysis of frost and drought tolerance inferred from Köppen–Geiger zones (Schat *et al.*, 2024, Preprint) and previous work suggesting that present-day grasses tend to be either frost or drought specialists (Visser *et al.*, 2014). These findings are also in line with the idea that there is a trade-off among abiotic stress responses, such that plants cannot be equally well adapted to multiple stressors, in particular both low temperature and drought (Puglielli *et al.*, 2021). Finally, our results mean that the evolutionary origins of shared genetic responses to cold and drought remain largely unknown. Gene expression patterns suggest some kind of shared ancestral response to both cold and drought in Pooideae (Schubert *et al.*, 2019a). These may have originated even further back in time, outside the Pooideae. Further research in a phylogenetic framework, with species sampled from across broad clades, will be needed to test this further.

Interestingly, we inferred the ancestor of Pooideae to have low frost tolerance, with higher levels being reconstructed only in core Pooideae (Triticeae and Poeae; Fig. 2). This contrasts with other reconstructions, suggesting frost tolerance at deeper nodes, for example as far back as the ancestor of all Pooideae except *Brachyelytrum* and *Nardus* plus *Lygeum* (Schubert *et al.*, 2019b; Schat *et al.*, 2024, Preprint). The use of an experimentally measured frost response here has thus provided a more nuanced view of how frost tolerance evolved in Pooideae, compared with studies relying on binary coding of this trait (frost sensitive/tolerant). Our reconstruction suggests frost tolerance increased only after transitions to open habitats occurred (Zhang *et al.*, 2022; Elliott *et al*., 2024) and coincidentally with the novel expansion of gene families involved in low temperature stress responses in core Pooideae (Sandve and Fjellheim, 2010; Schubert *et al.*, 2019a).

We found a higher phylogenetic signal to frost than drought tolerance (Table 2), that is, a higher signature of shared ancestry in frost tolerance than drought tolerance. This holds true even when the effects of phylogeny, geography, and the local climate are modelled together (Table 4), meaning it is not an artefact of not accounting for other potentially confounding variables (Freckleton and Jetz, 2009) (Humphreys and Linder, 2013; Coelho *et al.*, 2019; Lancaster and Humphreys, 2020). The high phylogenetic signal in frost tolerance is consistent with other studies in grasses (Edwards and Smith, 2010; Humphreys and Linder, 2013; Schat *et al.*, 2024, Preprint) and land plants (Lancaster and Humphreys, 2020) and suggests that rare evolutionary events structure frost responses in plants.

The low phylogenetic and geographical signals in drought responses (Tables 2, 4) could indicate that Pooideae rely on general stress tolerance mechanisms to cope with drought, rather than being drought specialists. Alternatively, the low phylogenetic signal suggests that drought tolerance is evolutionarily labile in Pooideae. This is supported by other similarly shallow reconstructions of drought tolerance in Pooideae, with xerophytes arising multiple times independently, only being reconstructed ancestrally in Triticeae (Zhang *et al.*, 2022), and drought tolerance being reconstructed at deeper ancestral nodes only in Stipeae and Triticeae (Schat *et al.*, 2024, Preprint). Our results together with these previous reconstructions suggest that origins of modern-day drought tolerance are decoupled from the transitions from closed to open habitats in Pooideae.

Evolutionary lability of drought tolerance is supported by other lines of evidence. Some form of drought tolerance is assumed to have been a key factor in the early evolution of land plants (Oliver *et al.*, 2000; Zhao *et al.*, 2019), but since then various forms of adaptations for avoiding dehydration have evolved, been lost, and been regained several times (Bowles *et al.*, 2021). Some gene families (e.g. *C-REPEAT BINDING FACTORS* (*CBFs*) and *dehydrins*) are expressed in response to a range of stressors, including drought, frost, and salinity (Chew and Halliday, 2011). These gene families are present in all plants at all times and are often larger and more flexible (undergoing expansions and contractions) than gene families not involved in stress responses (Chew and Halliday, 2011; Panchy *et al.*, 2016; Schubert *et al.*, 2019a). Such flexibility serves as a basis for adaptation, allowing individual genes to be co-opted into different stress responses in certain lineages (Schubert *et al.*, 2019a). If this is the genetic basis of drought tolerance in Pooideae, then this evolutionary lability will limit our ability to reconstruct the order in which drought tolerance traits evolved relative to other stress responses and assess their putative roles as evolutionary precursors (Bromham, 2014).

### Local climate conditions do not explain measured drought and frost responses

We found at best a weak correlation between experimentally measured drought and frost responses and aspects of the local climate in species’ native ranges (Table 3; Supplementary Dataset S2). There was no effect of the local climate for frost tolerance or leaf dry matter content but for drought tolerance there was a weak effect of mean annual temperature (bio1) and minimum temperature of the coldest month (bio6). These results indicate that the most drought tolerant species are from areas that are warm on average but with cold winters. For frost tolerance, phylogeny was the most important for explaining differences among species, but half of the variance was attributed to independent factors, suggesting that factors not included here may be important for explaining differences among species. Alternatively, the weak effect of climate or large variance partition attributed to independent factors may be due to the relatively small dataset analysed here. However, weak trait–environment relationships are consistent with previous findings for a range of plant traits (upper and lower thermal tolerances, plant height, leaf size, seed size; Moles *et al.*, 2014; but see Lancaster and Humphreys, 2020; Das *et al.*, 2021). In ours and previous studies, local temperature conditions explained more of the variation in functional or experimentally determined response variables than local precipitation conditions, but neither explained very much. This suggests that plants in general show only weak signatures of local adaptation and/or that air temperatures expressed by the BioClim variables at coarse geographical scales do not capture the (micro)climatic conditions plants are naturally exposed to (Greiser *et al.*, 2020). In support of the latter, land surface temperatures based on satellite measurements of radiative temperatures capture more differences in occupied thermal environments between closely related C_3_ and C_4_ grasses than air temperatures (Still *et al.*, 2014). Thus, we acknowledge some obvious limitations in characterizing species’ local climate conditions using coarse-grid BioClim variables. However, we also emphasize their demonstrated utility for macro-level studies. For example, annual averages can explain broadscale plant and trait distributions (*Zhao et al.*, 2019) and are widely used for defining ecoregions (Smith *et al*., 2018). Furthermore, previous large scale studies have shown that winter survival rates in grasses are higher for species from colder environments (Humphreys and Linder, 2013) and measured cold tolerances across land plants show the expected latitudinal pattern of increasing resistance with increasing latitude (Lancaster and Humphreys, 2020). This adds credibility to our findings here, that current climate conditions in species’ native ranges are only weakly related to measured frost and drought responses, with a greater role for evolutionary history in determining interspecific differences (at least of frost responses). Another factor not considered here is the stability of trait–environment relationships over time (Famiglietti *et al.*, 2023; Cui, 2024). Since our study concerns change in plant traits over evolutionary timescales, incorporating climate fluctuations through time should strengthen trait–environment relationships.

### Conclusion

We conclude that there is little evidence in our data for a positive correlation between drought and frost responses or that drought tolerance acted as a precursor to frost tolerance. Instead, our reconstructions suggest that present-day drought and frost responses are the result of independent evolutionary trajectories in different Pooideae lineages, or that their shared origins occurred outside the Pooideae. Either way, the evolutionary origins of the known physiological and genetic links between frost and drought responses remain unclear. Our results also suggest that origins of modern-day drought tolerance are decoupled from the transitions from closed to open habitats in Pooideae—but also that drought tolerance is more evolutionarily labile than frost tolerance. This lability could limit our ability to reconstruct the relative order in which drought and frost responses originated, potentially hampering assessment of their putative roles as evolutionary precursors. Further research is needed to discern whether our findings are unique to the cool season grasses, or whether signatures of shared evolutionary origins among diverse abiotic stress tolerance responses are no longer detectable on the long timescales studied here.

## Supplementary data

The following supplementary data are available at *JXB* online.

Table S1. Summary of the number of plants in the experiments.

Dataset S1. All data used in the analyses.

Dataset S2. Raw data from the experiments.

erae316_suppl_Supplementary_Table_S1

erae316_suppl_Supplementary_Dataset_S1

erae316_suppl_Supplementary_Dataset_S2

## Data Availability

The input for analyses can be found in Supplementary Dataset S1 and the raw data from the experiments can be found in Supplementary Dataset S2.

## References

[CIT0001] Akaike H. 1974. A new look at the statistical model identification. IEEE Transactions on Automatic Control 19, 716–723.

[CIT0002] Alm V, Busso CS, Ergon A, Rudi H, Larsen A, Humphreys MW, Rognli OA. 2011. QTL analyses and comparative genetic mapping of frost tolerance, winter survival and drought tolerance in meadow fescue (*Festuca pratensis* Huds.). Theoretical and Applied Genetics 123, 369–382. 21505831 10.1007/s00122-011-1590-z

[CIT0003] Anderson D, Burnham K. 2004. Model selection and multi-model inference. 2nd edn. New York: Springer-Verlag.

[CIT0004] Anisko T, Lindstrom OM. 1996. Cold hardiness and water relations parameters in *Rhododendron* cv. Catawbiense Boursault subjected to drought episodes. Physiologia Plantarum 98, 147–155.

[CIT0005] Bajji M, Kinet J-M, Lutts S. 2002. The use of the electrolyte leakage method for assessing cell membrane stability as a water stress tolerance test in durum wheat. Plant Growth Regulation 36, 61–70.

[CIT0006] Bennett TH, Flowers TJ, Bromham L. 2013. Repeated evolution of salt-tolerance in grasses. Biology Letters 9, 20130029. 23445947 10.1098/rsbl.2013.0029 PMC3639779

[CIT0007] Bouchenak-Khelladi Y, Verboom AG, Savolainen V, Hodkinson TR. 2010. Biogeography of the grasses (Poaceae): a phylogenetic approach to reveal evolutionary history in geographical space and geological time. Botanical Journal of the Linnean Society 162, 543–557.

[CIT0008] Bowles AMC, Paps J, Bechtold U. 2021. Evolutionary origins of drought tolerance in spermatophytes. Frontiers in Plant Science 12, 655924. 34239520 10.3389/fpls.2021.655924 PMC8258419

[CIT0009] Bromham L. 2014. Macroevolutionary patterns of salt tolerance in angiosperms. Annals of Botany 115, 333–341. 25452251 10.1093/aob/mcu229 PMC4332609

[CIT0010] Cardillo M, Skeels A. 2016. Spatial, phylogenetic, environmental and biological components of variation in extinction risk: a case study using *Banksia*. PLoS One 11, e0154431. 27148745 10.1371/journal.pone.0154431 PMC4858230

[CIT0011] Chew YH, Halliday KJ. 2011. A stress-free walk from *Arabidopsis* to crops. Current Opinion in Biotechnology 22, 281–286. 21168324 10.1016/j.copbio.2010.11.011

[CIT0012] Cialdella AM, Giussani LM, Aagesen L, Zuloaga FO, Morrone O. 2007. A phylogeny of *Piptochaetium* (Poaceae: Pooideae: Stipeae) and related genera based on a combined analysis including trnL-F, rpl16, and morphology. Systematic Botany 32, 545–559.

[CIT0013] Clifford D, McCullagh P. 2006. The regress function. R News 6, 6–10.

[CIT0014] Clifford D, McCullagh P. 2014. The Regress Package. R package version 1.3-15. https://github.com/kbroman/regress

[CIT0015] Coelho MTP, Rodrigues JFM, Diniz-Filho JAF, Rangel TF. 2019. Biogeographical history constrains climatic niche diversification without adaptive forces driving evolution. Journal of Biogeography 46, 1020–1028.

[CIT0016] Cornelissen JHC, Lavorel S, Garnier E, et al. 2003. A handbook of protocols for standardised and easy measurement of plant functional traits worldwide. Australian Journal of Botany 51, 335–380.

[CIT0017] Cui E. 2024. Trait–environment relationships are timescale dependent. New Phytologist 241, 2313–2315. 38263681 10.1111/nph.19546

[CIT0018] Das A, Dedon N, Enders DJ, Fjellheim S, Preston JC. 2023. Testing the chilling- before drought-tolerance hypothesis in Pooideae grasses. Molecular Ecology 32, 772–785. 36420966 10.1111/mec.16794 PMC10107940

[CIT0019] Das A, Prakash A, Dedon N, Doty A, Siddiqui M, Preston JC. 2021. Variation in climatic tolerance, but not stomatal traits, partially explains Pooideae grass species distributions. Annals of Botany 128, 83–95. 33772589 10.1093/aob/mcab046 PMC8318108

[CIT0020] Donoghue MJ. 2008. A phylogenetic perspective on the distribution of plant diversity. Proceedings of the National Academy of Sciences, USA 105, 11549–11555. 10.1073/pnas.0801962105 PMC2556411 18695216

[CIT0021] Ebermayer E. 1873. Die physikalischen einwirkungen des waldes auf luft und boden und seine klimatologische und hygienische bedeutung, begründet durch die beobachtungen der forst.-meteorolog. stationen im königreich Bayern. Aschaffenburg: C. Krebs.

[CIT0022] Edwards EJ, Osborne CP, Strömberg CAE, Smith SA, Consortium CG. 2010. The origins of C_4_ grasslands: integrating evolutionary and ecosystem science. Science 328, 587–591. 20431008 10.1126/science.1177216

[CIT0023] Edwards EJ, Smith SA. 2010. Phylogenetic analyses reveal the shady history of C_4_ grasses. Proceedings of the National Academy of Sciences, USA 107, 2532–2537. 10.1073/pnas.0909672107 PMC2823882 20142480

[CIT0024] Eldrett JS, Greenwood DR, Harding IC, Huber M. 2009. Increased seasonality through the Eocene to Oligocene transition in northern high latitudes. Nature 459, 969–973. 19536261 10.1038/nature08069

[CIT0025] Elliott TL, Spalink D, Larridon I, et al. 2024. Global analysis of Poales diversification – parallel evolution in space and time into open and closed habitats. New Phytologist 242, 727–743. 38009920 10.1111/nph.19421 PMC11497318

[CIT0026] Engelbrecht BMJ, Kursar TAJO. 2003. Comparative drought-resistance of seedlings of 28 species of co-occurring tropical woody plants. Oecologia 136, 383–393. 12811534 10.1007/s00442-003-1290-8

[CIT0027] Famiglietti CA, Worden M, Anderegg LDL, Konings AG. 2023. Impacts of climate timescale on the stability of trait–environment relationships. New Phytologist 241, 2423–2434. 38037289 10.1111/nph.19416

[CIT0028] Fick SE, Hijmans RJ. 2017. WorldClim 2: new 1-km spatial resolution climate surfaces for global land areas. International Journal of Climatology 37, 4302–4315.

[CIT0029] Fjellheim S, Boden S, Trevaskis B. 2014. The role of seasonal flowering responses in adaptation of grasses to temperate climates. Frontiers in Plant Science 5, 431. 25221560 10.3389/fpls.2014.00431 PMC4148898

[CIT0030] Fjellheim S, Young DA, Paliocha M, Johnsen SS, Schubert M, Preston JC. 2022. Major niche transitions in Pooideae correlate with variation in photoperiodic flowering and evolution of CCT domain genes. Journal of Experimental Botany 73, 4079–4093. 35394528 10.1093/jxb/erac149 PMC9232202

[CIT0031] Folk RA, Siniscalchi CM, Soltis DE. 2020. Angiosperms at the edge: Extremity, diversity, and phylogeny. Plant, Cell & Environment 43, 2871–2893. 10.1111/pce.13887 32926444

[CIT0032] Folk RA, Stubbs RL, Mort ME, Cellinese N, Allen JM, Soltis PS, Soltis DE, Guralnick RP. 2019. Rates of niche and phenotype evolution lag behind diversification in a temperate radiation. Proceedings of the National Academy of Sciences, USA 116, 10874–10882. 10.1073/pnas.1817999116 PMC6561174 31085636

[CIT0033] Freckleton RP, Jetz W. 2009. Space versus phylogeny: disentangling phylogenetic and spatial signals in comparative data. Proceedings of the Royal Society B: Biological Sciences 276, 21–30. 10.1098/rspb.2008.0905 PMC2614254 18796398

[CIT0034] Fujikawa S, Miura K. 1986. Plasma membrane ultrastructural changes caused by mechanical stress in the formation of extracellular ice as a primary cause of slow freezing injury in fruit-bodies of Basidiomycetes (*Lyophyllum ulmarium* (Fr.) Kühner). Cryobiology 23, 371–382.

[CIT0035] Gallaher TJ, Adams DC, Attigala L, Burke SV, Craine JM, Duvall MR, Klahs PC, Sherratt E, Wysocki WP, Clark LG. 2019. Leaf shape and size track habitat transitions across forest–grassland boundaries in the grass family (Poaceae). Evolution 73, 927–946. 30874302 10.1111/evo.13722

[CIT0036] Gallaher TJ, Peterson PM, Soreng RJ, Zuloaga FO, Li D-Z, Clark LG, Tyrrell CD, Welker CAD, Kellogg EA, Teisher JK. 2022. Grasses through space and time: An overview of the biogeographical and macroevolutionary history of Poaceae. Journal of Systematics and Evolution 60, 522–569.

[CIT0037] GBIF.org. 2022a. GBIF Occurrence Download. 10.15468/dl.26b2o8

[CIT0038] GBIF.org. 2022b. GBIF Occurrence Download. 10.15468/dl.3ybten

[CIT0039] GBIF.org. 2022c. GBIF Occurrence Download. 10.15468/dl.t3yqgc

[CIT0040] GBIF.org. 2022d. GBIF Occurrence Download. 10.15468/dl.w6a4dr

[CIT0041] Gilbert ME, Medina V. 2016. Drought adaptation mechanisms should guide experimental design. Trends in Plant Science 21, 639–647. 27090148 10.1016/j.tplants.2016.03.003

[CIT0042] Gillespie LJ, Archambault A, Soreng RJ. 2007. Phylogeny of *Poa* (Poaceae) based on *trn*T–*trn*F sequence data: major clades and basal relationships. Aliso 23, 420–434.

[CIT0043] Grebenstein B, Röser M, Sauer W, Hemleben V. 1998. Molecular phylogenetic relationships in *Aveneae* (Poaceae) species and other grasses as inferred from ITS1 and ITS2 rDNA sequences. Plant Systematics and Evolution 213, 233–250.

[CIT0044] Greiser C, Ehrlén J, Meineri E, Hylander K. 2020. Hiding from the climate: Characterizing microrefugia for boreal forest understory species. Global Change Biology 26, 471–483. 31833152 10.1111/gcb.14874 PMC7027894

[CIT0045] Hagen O, Vaterlaus L, Albouy C, Brown A, Leugger F, Onstein RE, de Santana CN, Scotese CR, Pellissier L. 2019. Mountain building, climate cooling and the richness of cold-adapted plants in the Northern Hemisphere. Journal of Biogeography 46, 1792–1807.

[CIT0046] Hamasha HR, von Hagen KB, Röser M. 2012. *Stipa* (Poaceae) and allies in the Old World: molecular phylogenetics realigns genus circumscription and gives evidence on the origin of American and Australian lineages. Plant Systematics and Evolution 298, 351–367.

[CIT0047] Harmon LJ, Weir JT, Brock CD, Glor RE, Challenger W. 2008. GEIGER: investigating evolutionary radiations. Bioinformatics 24, 129–131. 18006550 10.1093/bioinformatics/btm538

[CIT0048] Hartley W. 1973. Studies on the origin, evolution, and distribution of the Gramineae. V. The subfamily Festucoideae. Australian Journal of Botany 21, 201–234.

[CIT0049] Hincha DK, Höfner R, Schwab KB, Heber U, Schmitt JM. 1987. Membrane rupture is the common cause of damage to chloroplast membranes in leaves injured by freezing or excessive wilting. Plant Physiology 83, 251–253. 16665230 10.1104/pp.83.2.251 PMC1056342

[CIT0050] Hjertaas AC, Preston JC, Kainulainen K, Humphreys AM, Fjellheim S. 2023. Convergent evolution of the annual life history syndrome from perennial ancestors. Frontiers in Plant Science 13, 1048656. 36684797 10.3389/fpls.2022.1048656 PMC9846227

[CIT0051] Humphreys AM, Linder HP. 2013. Evidence for recent evolution of cold tolerance in grasses suggests current distribution is not limited by (low) temperature. New Phytologist 198, 1261–1273. 23528107 10.1111/nph.12244

[CIT0052] Hussain HA, Hussain S, Khaliq A, Ashraf U, Anjum SA, Men S, Wang L. 2018. Chilling and drought stresses in crop plants: implications, cross talk, and potential management opportunities. Frontiers in Plant Science 9, 393. 29692787 10.3389/fpls.2018.00393 PMC5902779

[CIT0053] Ives AR. 2018. *R*^2^s for correlated data: phylogenetic models, LMMs, and GLMMs. Systematic Biology 68, 234–251. 10.1093/sysbio/syy060 30239975

[CIT0054] Kellogg EA. 2001. Evolutionary history of the grasses. Plant Physiology 125, 1198–1205. 11244101 10.1104/pp.125.3.1198 PMC1539375

[CIT0055] Kellogg EA. 2015. Flowering plants, monocots: Poaceae. In: Kubitzki K, ed. The families and genera of vascular plants, Vol. 13. Cham, Switzerland: Springer International, 1–416.

[CIT0056] Kottek M, Grieser J, Beck C, Rudolf B, Rubel F. 2006. World map of the Köppen-Geiger climate classification updated. Meteorologische Zeitschrift 15, 259–263.

[CIT0057] Lancaster LT, Humphreys AM. 2020. Global variation in the thermal tolerances of plants. Proceedings of the National Academy of Sciences, USA 117, 13580–13587. 10.1073/pnas.1918162117 PMC7306813 32482870

[CIT0058] Larcher W. 2005. Climatic constraints drive the evolution of low temperature resistance in woody plants. Journal of Agricultural Meteorology 61, 189–202.

[CIT0059] Larsen A. 1978. Freezing tolerance in grasses—methods for testing in controlled environments. Meldinger fra Norges Landbrukshøgskole 57, 2–56.

[CIT0060] Lindberg CL, Hanslin HM, Schubert M, Marcussen T, Trevaskis B, Preston JC, Fjellheim S. 2020. Increased above-ground resource allocation is a likely precursor for independent evolutionary origins of annuality in the Pooideae grass subfamily. New Phytologist 228, 318–329. 32421861 10.1111/nph.16666

[CIT0061] Liu H, Xu Q, He P, Santiago LS, Yang K, Ye Q. 2015. Strong phylogenetic signals and phylogenetic niche conservatism in ecophysiological traits across divergent lineages of Magnoliaceae. Scientific Reports 5, 12246. 26179320 10.1038/srep12246 PMC4503962

[CIT0062] Liu Z, Pagani M, Zinniker D, Deconto R, Huber M, Brinkhuis H, Shah SR, Leckie RM, Pearson A. 2009. Global cooling during the Eocene-Oligocene climate transition. Science 323, 1187–1190. 19251622 10.1126/science.1166368

[CIT0063] McKeown M, Schubert M, Marcussen T, Fjellheim S, Preston JC. 2016. Evidence for an early origin of vernalization responsiveness in temperate Pooideae grasses. Plant Physiology 172, 416–426. 27474116 10.1104/pp.16.01023 PMC5074605

[CIT0064] Medeiros JS, Pockman WT. 2011. Drought increases freezing tolerance of both leaves and xylem of *Larrea tridentata*. Plant, Cell & Environment 34, 43–51. 10.1111/j.1365-3040.2010.02224.x 20825578

[CIT0065] Moles AT, Perkins SE, Laffan SW, et al. 2014. Which is a better predictor of plant traits: temperature or precipitation? Journal of Vegetation Science 25, 1167–1180.

[CIT0066] Monson RK, Rosenstiel TN, Forbis TA, Lipson DA, Jaeger CH III. 2006. Nitrogen and carbon storage in alpine plants. Integrative and Comparative Biology 46, 35–48. 21672721 10.1093/icb/icj006

[CIT0067] Monson RK, Smith SD. 1982. Seasonal water potential components of Sonoran desert plants. Ecology 63, 113–123.

[CIT0068] Oliver MJ, Tuba Z, Mishler BD. 2000. The evolution of vegetative desiccation tolerance in land plants. Plant Ecology 151, 85–100.

[CIT0069] Orme D, Freckleton R, Thomas G, Petzoldt T, Fritz S, Isaac N, Pearse W. 2018. caper: comparative analyses of phylogenetics and evolution in R. R package version 1.0.1. https://CRAN.R-project.org/package=caper

[CIT0070] Pagel M. 1999. Inferring the historical patterns of biological evolution. Nature 401, 877–884. 10553904 10.1038/44766

[CIT0071] Panchy N, Lehti-Shiu MD, Shiu S-H. 2016. Evolution of gene duplication in plants. Plant Physiology 171, 2294–2316. 27288366 10.1104/pp.16.00523 PMC4972278

[CIT0072] Paradis E, Schliep K. 2018. ape 5.2: an environment for modern phylogenetics and evolutionary analyses in R. Bioinformatics 35, 526–528. 10.1093/bioinformatics/bty633 30016406

[CIT0073] Pearce RS. 2001. Plant freezing and damage. Annals of Botany 87, 417–424.

[CIT0074] Pescador DS, Sierra-Almeida A, Torres PJ, Escudero A. 2016. Summer freezing resistance: a critical filter for plant community assemblies in Mediterranean high mountains. Frontiers in Plant Science 7, 194. 26941761 10.3389/fpls.2016.00194 PMC4761790

[CIT0075] Pisek A, Larcher W. 1954. Zusammenhang zwischen austrocknungsresistenz und frosthärte bei immergrünen. Protoplasma 44, 30–46.

[CIT0076] Pound MJ, Salzmann U. 2017. Heterogeneity in global vegetation and terrestrial climate change during the late Eocene to early Oligocene transition. Scientific Reports 7, 43386. 28233862 10.1038/srep43386 PMC5324063

[CIT0077] Preston JC, Sandve SR. 2013. Adaptation to seasonality and the winter freeze. Frontiers in Plant Science 4, 167. 23761798 10.3389/fpls.2013.00167 PMC3669742

[CIT0078] Puglielli G, Hutchings MJ, Laanisto L. 2021. The triangular space of abiotic stress tolerance in woody species: a unified trade-off model. New Phytologist 229, 1354–1362. 32989754 10.1111/nph.16952

[CIT0079] R Core Team. 2018. R: a language and environment for statistical computing. Vienna, Austria: R Foundation for Statistical Computing.

[CIT0080] Ricklefs RE, Renner SS. 1994. Species richness within families of flowering plants. Evolution 48, 1619–1636. 28568402 10.1111/j.1558-5646.1994.tb02200.x

[CIT0081] Rogers A, Dietz K-J, Gifford ML, Lunn JE. 2021. The importance of independent replication of treatments in plant science. Journal of Experimental Botany 72, 5270–5274. 34320198 10.1093/jxb/erab268

[CIT0082] RStudio Team. 2016. RStudio: integrated development for R. Boston, MA: RStudio PBC.

[CIT0083] Sakai A, Larcher W. 1987. Frost survival of plants: responses and adaptation to freezing stress. Berlin, Heidelberg: Springer Verlag.

[CIT0084] Sandve SR, Fjellheim S. 2010. Did gene family expansions during the Eocene–Oligocene boundary climate cooling play a role in Pooideae adaptation to cool climates? Molecular Ecology 19, 2075–2088. 20406386 10.1111/j.1365-294X.2010.04629.x

[CIT0085] Sandve SR, Kosmala A, Rudi H, Fjellheim S, Rapacz M, Yamada T, Rognli OA. 2011. Molecular mechanisms underlying frost tolerance in perennial grasses adapted to cold climates. Plant Science 180, 69–77. 21421349 10.1016/j.plantsci.2010.07.011

[CIT0086] Schat L, Schubert M, Fjellheim S, Humphreys AM. 2024. Drought tolerance as an evolutionary precursor to frost and winter tolerance in grasses. bioRxiv 2024.2006.2029.601311 [Preprint]. 10.1093/evolut/qpaf006 39826096

[CIT0087] Schubert M, Grønvold L, Sandve SR, Hvidsten TR, Fjellheim S. 2019a. Evolution of cold acclimation and its role in niche transition in the temperate grass subfamily Pooideae. Plant Physiology 180, 404–419. 30850470 10.1104/pp.18.01448 PMC6501083

[CIT0088] Schubert M, Humphreys AM, Lindberg CL, Preston JC, Fjellheim S. 2020. To coldly go where no grass has gone before: a multidisciplinary review of cold adaptation in Poaceae. Annual Plant Reviews Online 2020, 523–562.

[CIT0089] Schubert M, Marcussen T, Meseguer AS, Fjellheim S. 2019b. The grass subfamily Pooideae: Cretaceous–Palaeocene origin and climate-driven Cenozoic diversification. Global Ecology and Biogeography 28, 1168–1182.

[CIT0090] Shinozaki K, Yamaguchi-Shinozaki K. 2000. Molecular responses to dehydration and low temperature: differences and cross-talk between two stress signaling pathways. Current Opinion in Plant Biology 3, 217–223. 10837265

[CIT0091] Shinozaki K, Yamaguchi-Shinozaki K, Seki M. 2003. Regulatory network of gene expression in the drought and cold stress responses. Current Opinion in Plant Biology 6, 410–417. 12972040 10.1016/s1369-5266(03)00092-x

[CIT0092] Sierra-Almeida A, Reyes-Bahamonde C, Cavieres LA. 2016. Drought increases the freezing resistance of high-elevation plants of the Central Chilean Andes. Oecologia 181, 1011–1023. 27053321 10.1007/s00442-016-3622-5

[CIT0093] Smith JR, Letten AD, Ke PJ, et al. 2018. A global test of ecoregions. Nature Ecology and Evolution 2, 1889–1896. 30397301 10.1038/s41559-018-0709-x

[CIT0094] Soreng RJ, Peterson PM, Romaschenko K, Davidse G, Teisher JK, Clark LG, Barberá P, Gillespie LJ, Zuloaga FO. 2017. A worldwide phylogenetic classification of the Poaceae (Gramineae) II: an update and a comparison of two 2015 classifications. Journal of Systematics and Evolution 55, 259–290.

[CIT0095] Soreng RJ, Peterson PM, Romaschenko K, Davidse G, Zuloaga FO, Judziewicz EJ, Filgueiras TS, Davis JI, Morrone O. 2015. A worldwide phylogenetic classification of the Poaceae (Gramineae). Journal of Systematics and Evolution 53, 117–137.

[CIT0096] Stevens PF. 2017. Angiosperm Phylogeny Website. Version 14, July 2017. http://www.mobot.org/MOBOT/research/APweb/

[CIT0097] Still CJ, Pau S, Edwards EJ. 2014. Land surface skin temperature captures thermal environments of C_3_ and C_4_ grasses. Global Ecology and Biogeography 23, 286–296.

[CIT0098] Streeter JG, Lohnes DG, Fioritto RJ. 2001. Patterns of pinitol accumulation in soybean plants and relationships to drought tolerance. Plant, Cell & Environment 24, 429–438.

[CIT0099] Strömberg CAE. 2011. Evolution of grasses and grassland ecosystems. Annual Review of Earth and Planetary Sciences 39, 517–544.

[CIT0100] Sumner EE, Williamson VG, Gleadow RM, Wevill T, Venn SE. 2022. Acclimation to water stress improves tolerance to heat and freezing in a common alpine grass. Oecologia 199, 831–843. 35974110 10.1007/s00442-022-05245-1 PMC9464112

[CIT0101] Taiz L, Zeiger E, Møller IM, Murphy AS. 2015. Plant physiology and development. Sunderland, MA, USA: Sinauer Associates Incorporated.

[CIT0102] The Plant List. 2013. Version 1.1. http://www.theplantlist.org

[CIT0103] Vavrek M. 2011. *fossil*: palaeoecological and palaeogeographical analysis tools. Palaeontologia Electronica 14, 1T:16p.

[CIT0104] Vicre M, Farrant JM, Driouich A. 2004. Insights into the cellular mechanisms of desiccation tolerance among angiosperm resurrection plant species. Plant, Cell & Environment 27, 1329–1340.

[CIT0105] Visser V, Clayton WD, Simpson DA, Freckleton RP, Osborne CP. 2014. Mechanisms driving an unusual latitudinal diversity gradient for grasses. Global Ecology and Biogeography 23, 61–75.

[CIT0106] Vu VQ. 2011. ggbiplot: a ggplot2 based biplot. R package version 0.55. https://cran.r-project.org/web/packages/ggbiplot

[CIT0107] Watcharamongkol T. 2019. Migration to colder climates in grasses involves pre-existing and adaptive traits. PhD thesis, University of Sheffield.

[CIT0108] Watcharamongkol T, Christin P-A, Osborne CP. 2018. C_4_ photosynthesis evolved in warm climates but promoted migration to cooler ones. Ecology Letters 21, 376–383. 29318753 10.1111/ele.12905

[CIT0109] Wickham H. 2016. ggplot2: elegant graphics for data analysis. New York: Springer-Verlag New York.

[CIT0110] Wilke CO. 2019. cowplot: streamlined plot theme and plot annotations for ‘ggplot2’. R package version 0.9.4. https://cran.r-project.org/web/packages/cowplot/index.html

[CIT0111] Yu G, Smith D, Zhu H, Guan Y, Lam TT-Y. 2017. ggtree: an R package for visualization and annotation of phylogenetic trees with their covariates and other associated data. Methods in Ecology and Evolution 8, 28–36.

[CIT0112] Zachos J, Pagani M, Sloan L, Thomas E, Billups K. 2001. Trends, rhythms, and aberrations in global climate 65 Ma to present. Science 292, 686–693. 11326091 10.1126/science.1059412

[CIT0113] Zhang L, Zhu X, Zhao Y, Guo J, Zhang T, Huang W, Huang J, Hu Y, Huang C-H, Ma H. 2022. Phylotranscriptomics resolves the phylogeny of Pooideae and uncovers factors for their adaptive evolution. Molecular Biology and Evolution 39, msac026. 35134207 10.1093/molbev/msac026 PMC8844509

[CIT0114] Zhao C, Wang Y, Chan KX, et al. 2019. Evolution of chloroplast retrograde signaling facilitates green plant adaptation to land. Proceedings of the National Academy of Sciences, USA 116, 5015–5020. 10.1073/pnas.1812092116 PMC6421419 30804180

[CIT0115] Zhong J, Robbett M, Poire A, Preston JC. 2017. Successive evolutionary steps drove Pooideae grasses from tropical to temperate regions. New Phytologist 217, 925–938. 29091285 10.1111/nph.14868

