# Supplementary material for: Evolution of drought and frost responses in cool season grasses (Pooideae): was drought tolerance a precursor to frost tolerance?
Source: J Exp Bot. 2024 Jul 27;75(20):6405–22. doi: 10.1093/jxb/erae316 (PMC11522984; doi:10.1093/jxb/erae316)
Supplement: erae316_suppl_Supplementary_Table_S1 [file erae316_suppl_supplementary_table_s1.pdf]

Table S1. Number of individuals per species in each treatment group. The number is the experimental population number from Table 1.

Number	Species	Control	Drought	Sudden frost -1	Sudden frost -3	Weight	Start conductivity	Total
SR3	Poa trivialis	10	10	10	10	4	4	**48**
SR4	Deschampsia cespitosa	10	7	9	9	4	4	**43**
SR5	Poa alpina	11	10	10	10	4	4	**49**
SR6	Phleum alpinum	10	10	10	10	4	4	**48**
SR7	Lolium perenne	10	10	10	10	4	3	**47**
SR8	Dactylis glomerata	10	10	10	10	4	4	**48**
SR9	Poa alopecurus	5	8	9	9	4	4	**39**
SR10	Poa bulbosa	10	10	10	9	4	4	**47**
SR11	Festuca pratensis	5	9	9	8	4	4	**39**
SR13	Sesleria autumnalis	10	10	10	10	4	4	**48**
SR14	Vulpia myuros	10	10	10	10	4	4	**48**
SR15	Phleum pratense	10	10	10	10	4	4	**48**
SR16	Puccinellia distans	10	10	10	10	4	4	**48**
SR17	Festuca rubra	9	10	10	10	4	4	**47**
SR18	Festuca arundinacea	10	10	10	10	4	4	**48**
SR19	Phleum pratense	10	10	10	10	4	4	**48**
SR20	Holcus lanatus	10	10	10	10	4	4	**48**
SR21	Festuca ovina	10	10	10	10	4	4	**48**
SR22	Cynosurus cristatus	10	10	10	10	4	4	**48**
SR23	Alopecurus pratensis	10	10	10	10	4	4	**48**
SR24	Lolium multiflorum	10	10	10	10	4	4	**48**
SR25	Deschampsia atropurpurea	9	10	10	10	4	4	**47**
SR26	Poa glauca	10	10	10	10	4	4	**48**
SR28	Anthoxanthum odoratum	10	10	10	10	4	4	**48**
SR29	Phalaris arundinacea	10	10	10	10	4	4	**48**
SR30	Calamagrostis purpurea	10	10	10	10	4	4	**48**
SR31	Agrostis canina	10	10	10	10	4	4	**48**
SR32	Polypogon viridis	7	8	8	8	4	4	**39**
SR33	Helictotrichon pratense	10	9	10	10	4	4	**47**
SR35	Koeleria glauca	10	9	10	10	4	4	**47**
SR36	Trisetum flavescens	10	10	10	10	4	4	**48**
SR37	Briza minor	10	10	10	10	4	4	**48**
SR38	Briza media	10	10	10	10	4	4	**48**
SR39	Agrostis capillaris	10	10	10	10	4	4	**48**
SR40	Trisetum spicatum	8	10	10	10	4	4	**46**
SR41	Agrostis mertensii	5	5	6	6	4	3	**29**
SR43	Elymus repens	10	10	10	10	4	4	**48**
SR44	Triticum turgidum	10	10	10	10	4	4	**48**
SR45	Aegilops triuncialis	10	10	10	10	4	4	**48**
SR47	Hystrix patula	10	10	10	10	4	4	**48**
SR48	Hordeum jubatum	10	10	10	10	4	4	**48**
SR50	Dasypyrum villosum	10	10	10	10	4	4	**48**
SR52	Agropyron cristatum	6	9	9	9	4	3	**40**
SR54	Brachypodium pinnatum	10	10	10	10	4	4	**48**
SR57	Melica nutans	10	10	10	10	4	4	**48**
SR58	Glyceria striata	10	10	10	10	4	4	**48**
SR61	Glyceria occidentalis	10	10	10	10	4	4	**48**

SR62	Nassella hyalina	10	10	10	10	4	4	**48**
SR64	Stipa capillata	10	10	10	10	4	4	**48**
SR65	Stipa pekinense	10	10	10	10	4	4	**48**
SR66	Stipa gigantea	10	10	10	10	4	4	**48**
SR67	Stipa ichu	10	10	10	10	4	4	**48**
SR70	Nassella tenuissima	9	9	9	9	4	4	**44**
SR71	Nassella trichotoma	10	10	10	10	4	4	**48**
SR73	Piptochaetium fimbriatum	6	6	6	6	4	4	**32**
SR79	Nassella cernua	10	10	10	10	4	4	**48**
SR82	Stipa calamagrostis	10	10	10	10	4	4	**48**
SR89	Stipa caragana	6	9	9	9	4	4	**41**
SR92	Lygeum spartum	5	8	8	8	4	4	**37**
SR99	Nassella pubiflora	10	10	10	10	4	4	**48**
SR100	Melica ciliata	10	9	10	10	4	3	**46**
SR101	Piptatherum miliaceum	10	10	10	10	4	4	**48**
**Total**		**581**	**595**	**602**	**600**	**248**	**244**	**2870**
